# Supplementary material for: Dynamic global acetylation remodeling during the yeast heat shock response
Source: Genome Biol. 2026 Jul 17;27:229. doi: 10.1186/s13059-026-04194-9 (PMC13378067; doi:10.1186/s13059-026-04194-9)
Supplement: Supplementary file 1 — Additional file 1: Supplementary Figs S1-S6. [file 13059_2026_4194_MOESM1_ESM.pdf]

Supplemental Figures

Dynamic global acetylation remodeling during the yeast heat shock response

Rebecca E. Hardman-Kavanaugh[1,2], Aaron J. Storey[3], Tara N. Stuecker[2], Stephanie E. Hood[2], Gregory A. Barrett-Wilt[4], Venkata R. Krishnamurthi[5], Yong Wang[1, 5, 6], Stephanie D. Byrum[3], Samuel G. Mackintosh[3], Rick D. Edmondson[7], Wayne P. Wahls[3], Alan J. Tackett[3], and Jeffrey A. Lewis[2,*]

[1] Interdisciplinary Graduate Program in Cell and Molecular Biology, University of Arkansas, Fayetteville, Arkansas 72701, United States of America

[2] Department of Biological Sciences, University of Arkansas, Fayetteville, Arkansas 72701, United States of America

[3] Department of Biochemistry and Molecular Biology, University of Arkansas for Medical Sciences, Little Rock, Arkansas 72205, United States of America

[4] Biotechnology Center, University of Wisconsin, Madison, WI, 53706, United States of America

[5] Department of Physics, University of Arkansas, Fayetteville 72701, AR, United States of America

[6] Materials Science and Engineering Program, University of Arkansas, Fayetteville 72701, AR, United States of America

[7] College of Medicine, University of Arkansas for Medical Sciences, Little Rock, Arkansas 72205, United States of America

* Corresponding author

Jeffrey A. Lewis
Department of Biological Sciences
University of Arkansas
850 W. Dickson St., SCEN 601 Fayetteville, AR
72701
E-mail: lewisja@uark.edu
Tel: +1.479.575.7740

`

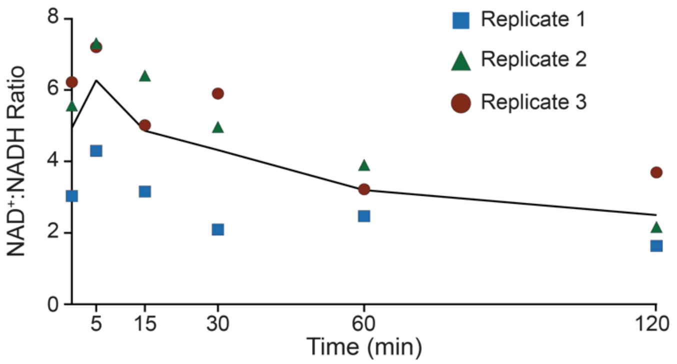

Additional file 1: Fig. S1. The NAD$^+$:NADH ratio initially spikes and then gradually decreases during heat shock. The NAD$^+$:NADH ratio was monitored across a 120 min heat shock (25°C to 37°C) via a luminescence assay.

`

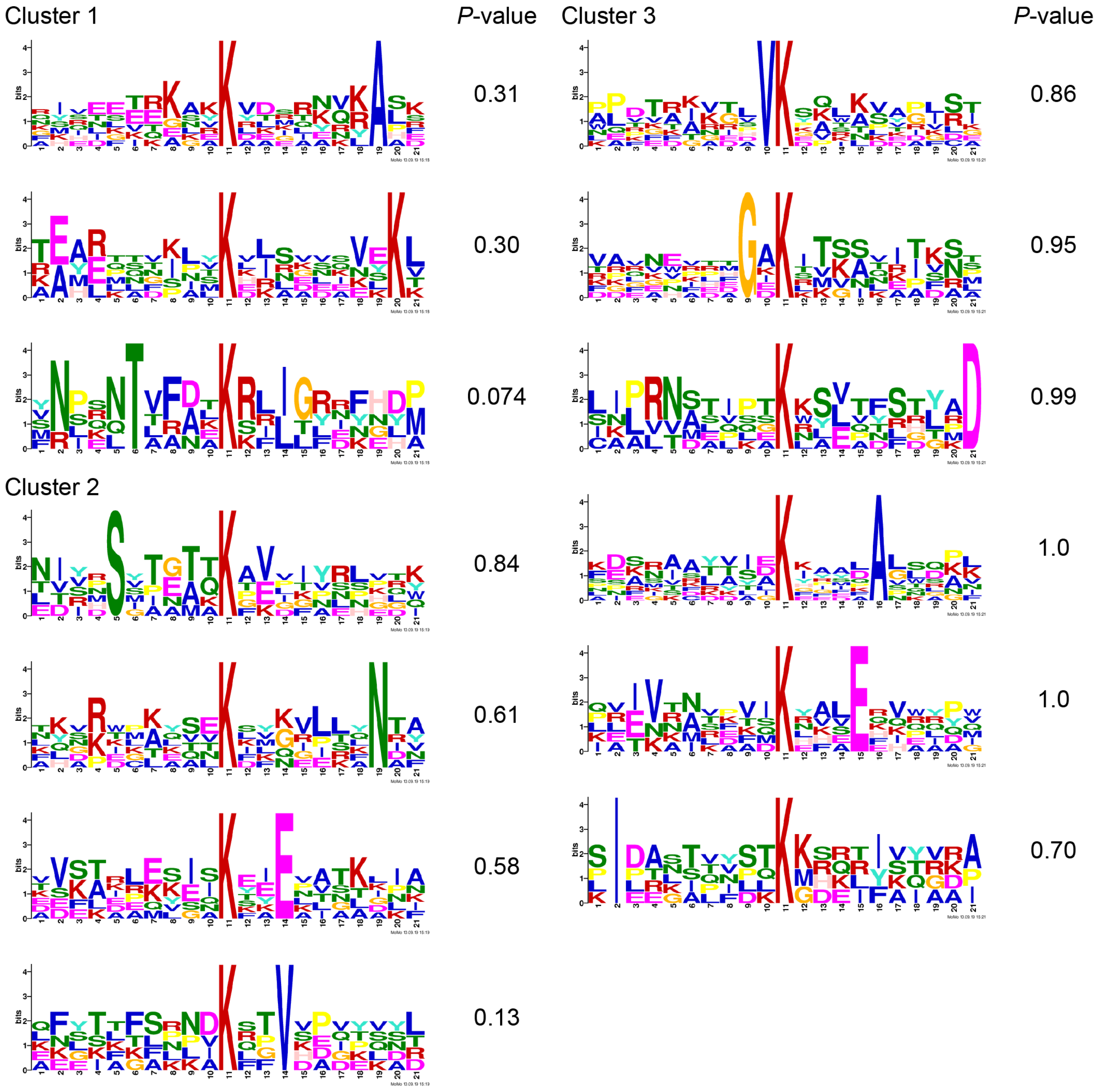

Additional file 1: Fig. 2. Motif analysis of clusters with increasing acetylation. Motif analysis of Clusters 1-3 from Figure 5A was performed using MoMo.

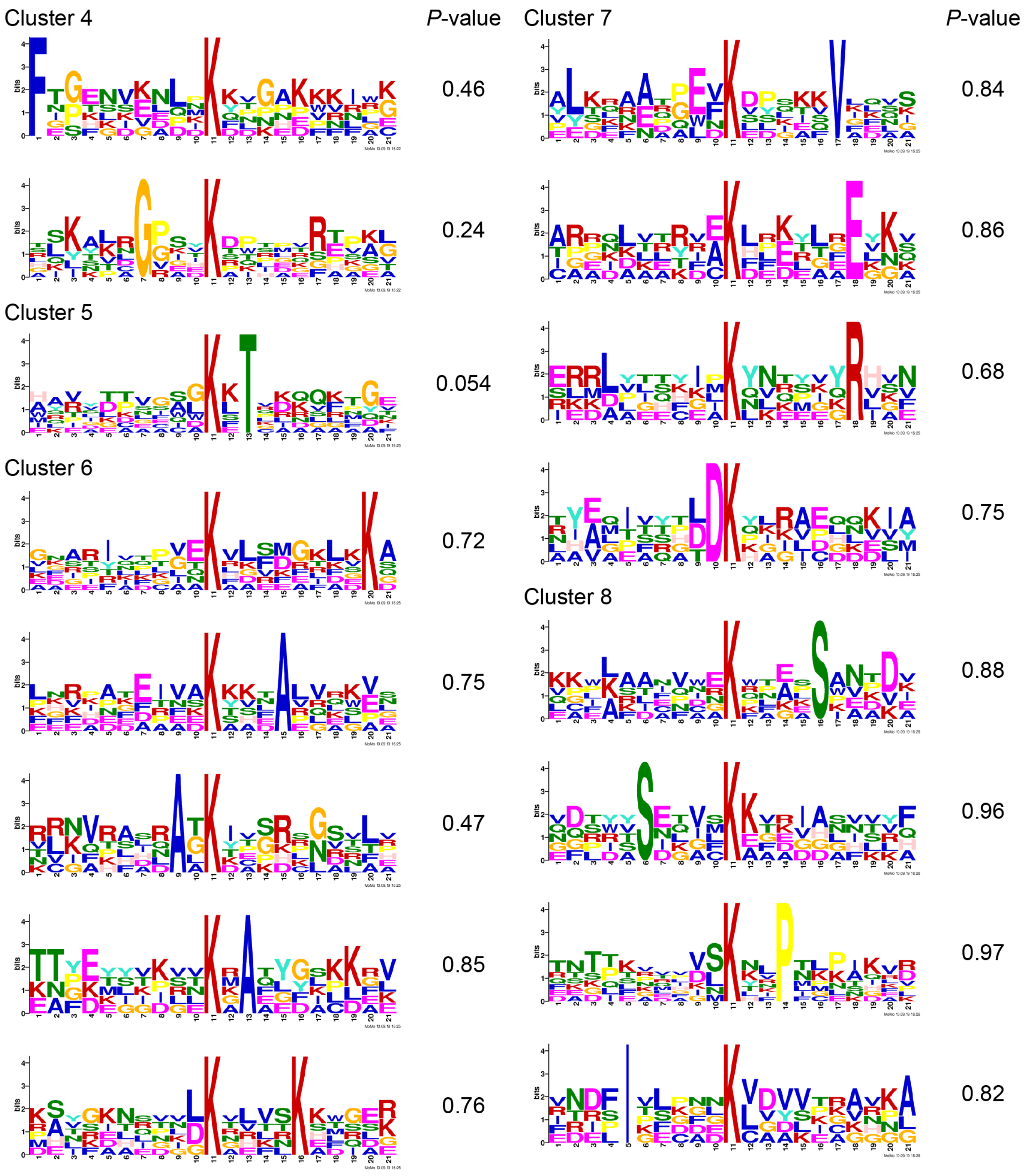

Additional file 1: Fig. S3. Motif analysis of clusters with decreasing acetylation. Motif analysis of Clusters 4-8 from Figure 5A was performed using MoMo.

`

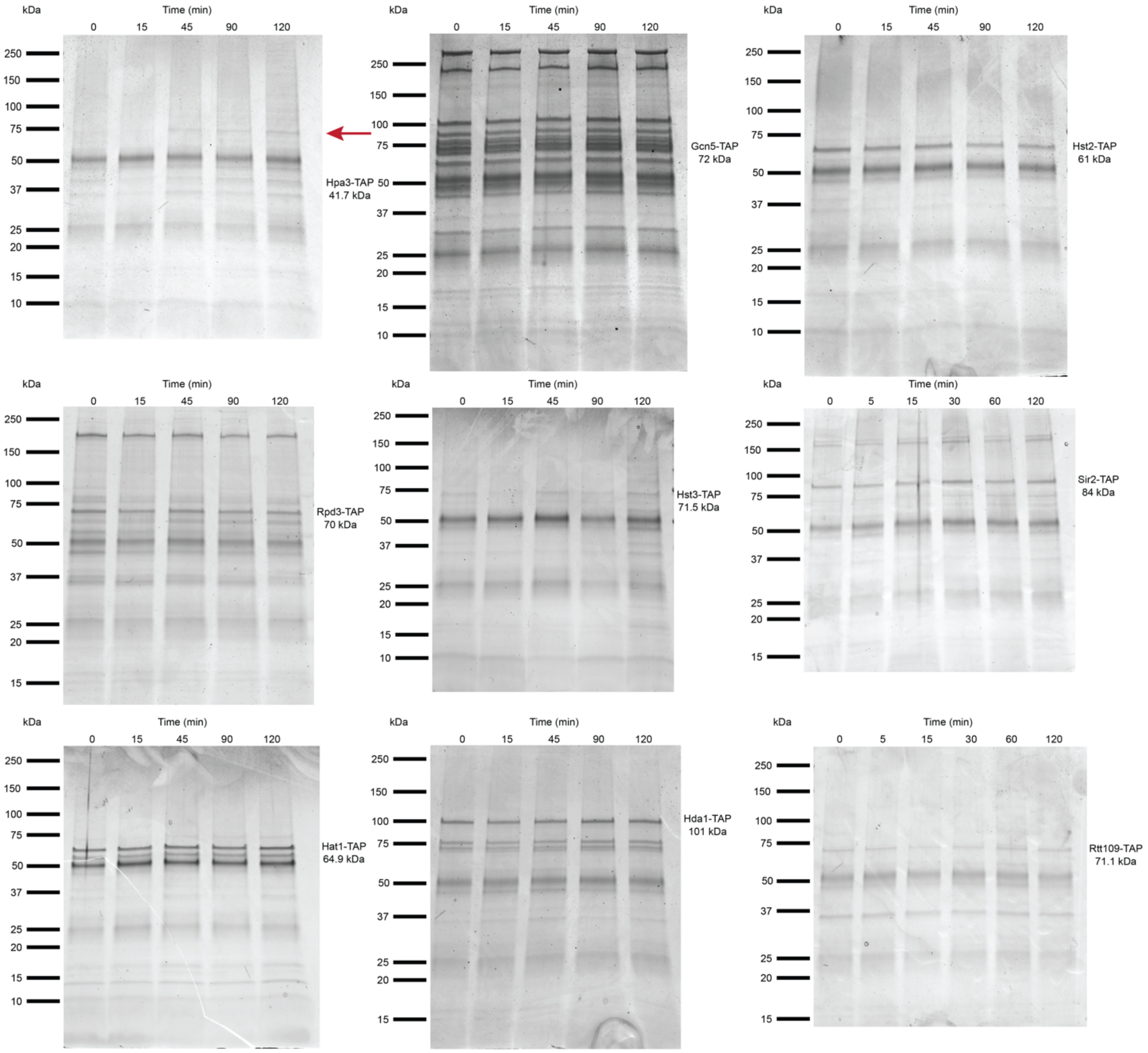

Additional file 1: Fig. S4. Tap co-immunoprecipitations of KAT and KDAC enzymes and interacting proteins. Identified KAT and KDAC enzymes and interacting proteins were co-immunoprecipitated and visualized with blue-silver staining. One interacting protein changed in abundance for the Hpa3 co-immunoprecipitation (red arrow), but could not be identified. No changes were observed for the other KATs and KDACs shown in the figure.

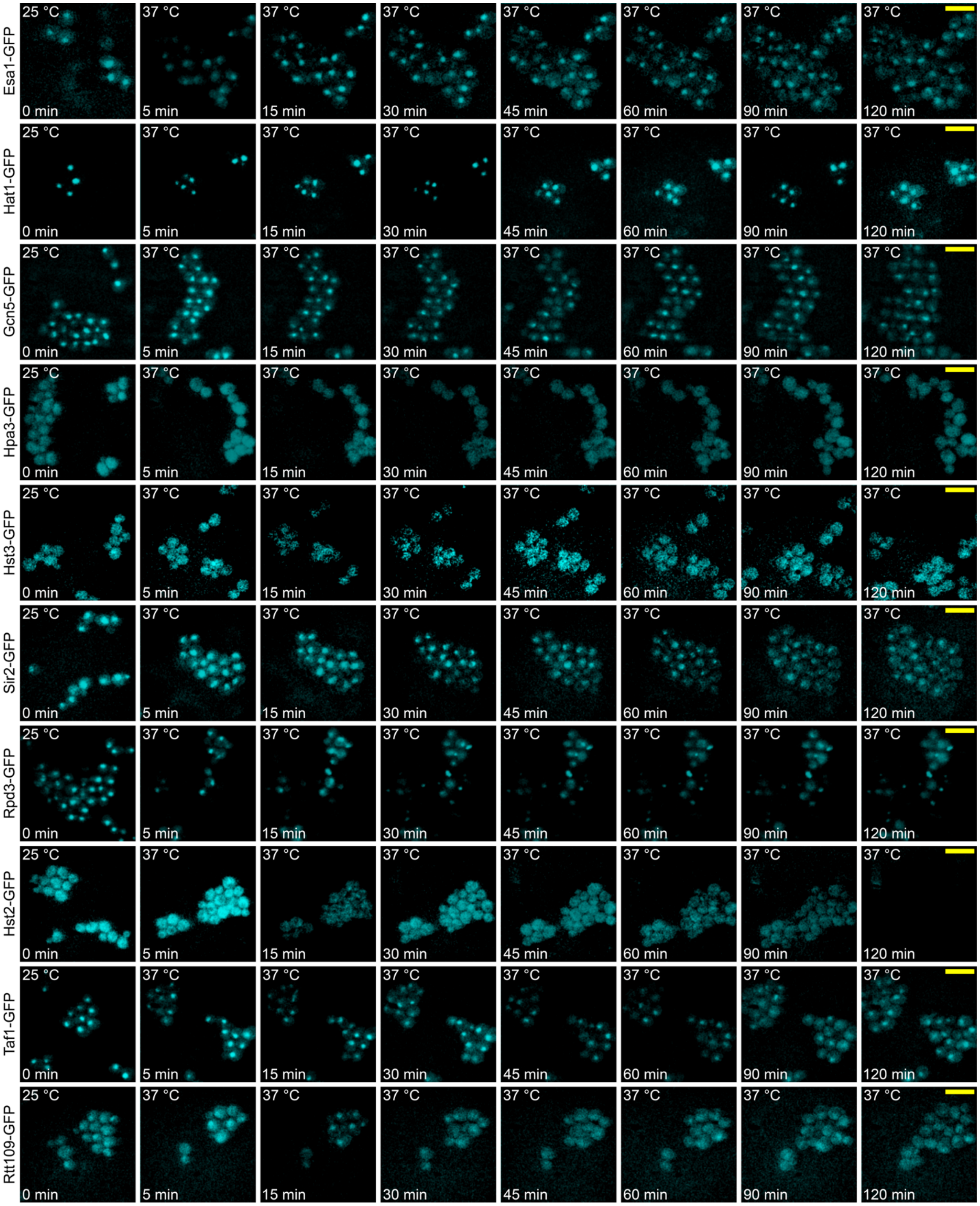

Additional file 1: Fig. S5. Fluorescence microscopy of KATs and KDACs. Strains carrying the depicted KAT or KDAC-GFP fusions were visualized at 37°C for 2 hours to determine possible changes in localization. None of the depicted proteins show changes in localization.

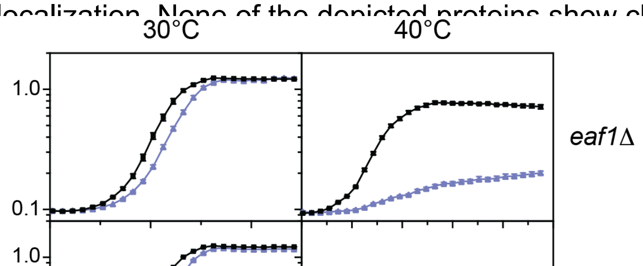

Additional file 1: Fig. S6. Growth phenotypes of KAT and KDAC mutants or their suspected dynamic interaction partners during heat shock. Growth assays were performed at 40°C comparing wild-type cells with the depicted mutants. The *sir2*Δ mutant was a MATa haploid, and was compared to haploid BY4741. All other mutants were homozygous diploids and were compared to the diploid BY4743. Error bars denote the standard error of 3 biological replicates.
